# Magnetic response of Arsenic pollution in a slag covered soil profile close to an abandoned tungsten mine, southern China

**DOI:** 10.1038/s41598-020-61411-6

**Published:** 2020-03-09

**Authors:** Tingping Ouyang, Mingkun Li, Erwin Appel, Zhihua Tang, Shasha Peng, Sang Li, Zhaoyu Zhu

**Affiliations:** 1 School of Geography, South China Normal University, Guangzhou, 510631 China; 2 Key Laboratory of Ocean and Marginal Sea Geology, Guangzhou Institute of Geochemistry, Chinese Academy of Sciences, Guangzhou, 510640 China; 3 Department of Geosciences, University of Tübingen, Hölderlinstrasse 12, 72074 Tübingen, Germany; 4 Guangzhou Institute of Energy Conversion, Chinese Academy of Sciences, Guangzhou, 510640 China

**Keywords:** Environmental sciences, Solid Earth sciences, Geochemistry, Geomagnetism

## Abstract

Previous studies indicated serious soil arsenic (As) pollution of large spatial extent related to tungsten mining. We performed systematic analyses of magnetic parameters and As contents of a slag covered soil profile close to the abandoned tungsten mine in southern China, in order to discuss the feasibility of using sensitive, non-destructive, and cost-effective magnetic methods for monitoring the soil arsenic content in such arsenic pollution areas. The results indicate that arsenic sulfide entered from slags into the underlying soil and changed to iron arsenate and moveable arsenic ion. The arsenic ions were transported from the upper to the lower part of the soil profile, leading to more serious arsenic pollution at lower levels of the section. Pedogenesis and oxidation of the entered iron and arsenic sulfide resulted in coexistence of magnetite/maghemite and hematite, with different contributions at depths of 125–195 cm, 60–125 cm, and 0–60 cm. The arsenic content is significant positively correlated with the hematite concentration given by the magnetic parameter HIRM and negatively correlated with the S_−300_ ratio that measures the relative contributions of magnetite(+maghemite) and hematite. The S_−300_ ratio is effective for semi-quantification of soil arsenic content, and may be also used for soil arsenic pollution assessment and monitoring in similar settings of tungsten mining.

## Introduction

Soil environment has been becoming one of the hottest issues of environmental science as well as environmental protection agency. As well known, mine utilization usually seriously affects the surrounding soil environment. Particularly, exploitation of metallic ore always leads to heavy metal pollution^1–3^. Serious heavy metal pollution frequently appeared worldwide^4–7^. Previous studies indicated that both surface and deep soils within and around metallic mine area such as the Lianhuashan abandoned tungsten mine at south China are being experienced serious arsenic pollution^8,9^. According to the “Law of the People’s Republic of China on the prevention and control of soil pollution”, soils within and around thus mine area are the key monitoring objects of heavy metal pollution. Therefore, rapid monitoring and identification of heavy metal pollution in soil within and around mining areas are a necessary prerequisite for mine environmental impact assessment and repair of mining damages. Rapid and effective methods are urgently needed for heavy metal content monitoring and pollution assessment.

Environmental magnetic methods have several advantages such as rapidness, sensitiveness, non-destructiveness, and cost-effectiveness^10,11^. Magnetic approaches were widely applied for soil heavy metal content monitoring and pollution assessment during the last decades^12–15^. Many studies indicated significant positive correlations between soil magnetic parameters (e.g., magnetic susceptibility, MS) and heavy metal contents. Thus, in some studies, MS was regarded as an effective indicator for heavy metal contamination in soil^16–18^. However, direct application of environmental magnetism in heavy metal monitoring and pollution assessment is limited due to the unclear key issue of and the underlying mechanisms causing the relationship between magnetic particles and heavy metal elements.

Previous studies verified that arsenic is often absorbed by iron oxides and oxyhydroxides, which form the most important and widely distributed magnetic minerals such as magnetite, hematite and goethite within soils^19–21^. Furthermore, soil magnetism can be elevated with increasing arsenic content due to containing arsenic in magnetic mineral structure^22^. Therefore, magnetic properties have the potential to detect arsenic concentration in soils. In order to reveal the coexistence mechanism of arsenic and magnetic particles and the feasibility of using magnetic parameters to monitor arsenic in polluted soil, an arsenic sulfide-rich slag covered soil profile close to an abandoned tungsten mine in southern China was selected for magnetic properties and arsenic content measurements in the present study. The main purpose of this study is to reveal the magnetic response of soil arsenic pollution and the mechanism for coexistence of arsenic and magnetic minerals. Moreover, we try to investigate not only qualitative relationship but also quantitative calculation equations between soil arsenic and magnetic parameters. Based on the mentioned results, effective magnetic parameters are suggested for soil arsenic content monitoring and quantifying pollution in such mine areas.

## Materials and methods

### Study area and sampling

As one of the biggest tungsten mines in Guangdong Province, southern China, the Lianhuashan tungsten mine area (23°38′30,4″N, 116°50′4.7″E) covers 2.122 km^2^. The combination of mineral deposits is characterized by the coexistence of wolframite and scheelite, rich in sulfides, especially pyrite, arsenopyrite, etc. These sulfides are rich in heavy metals such as Cu, Co, Ni, As^23–27^. Subtropical monsoon climate characterized by a wet and hot summer and a dry and cool winter, with mean annual temperature of 21.1 °C and mean annual rainfall of 1444 mm predominates this area. The tungsten mine was exploited since 1957. Due to the depletion of resources, mining was stopped in 1991 and the mine was closed in 1999. Since no effective protection and treatment measures were taken during the period of mining activities and after sealing the mine, the area affected by mine development and utilization exceeded 200 km^2^. Previous researches indicated that soil within and around the mine area is seriously polluted by heavy metals especially As^28–30^. According to X-ray diffraction (XRD) results for tens of surface soil samples around the mine area (unpublished data), the main minerals within surface soil in this area are quartz (about 40–50%), clay minerals (about 30–40%, including illite, montmorillonite, and kaolinite), and some other minerals such as gypsum, pyrite, magnetite, hematite.

**Figure 1:**
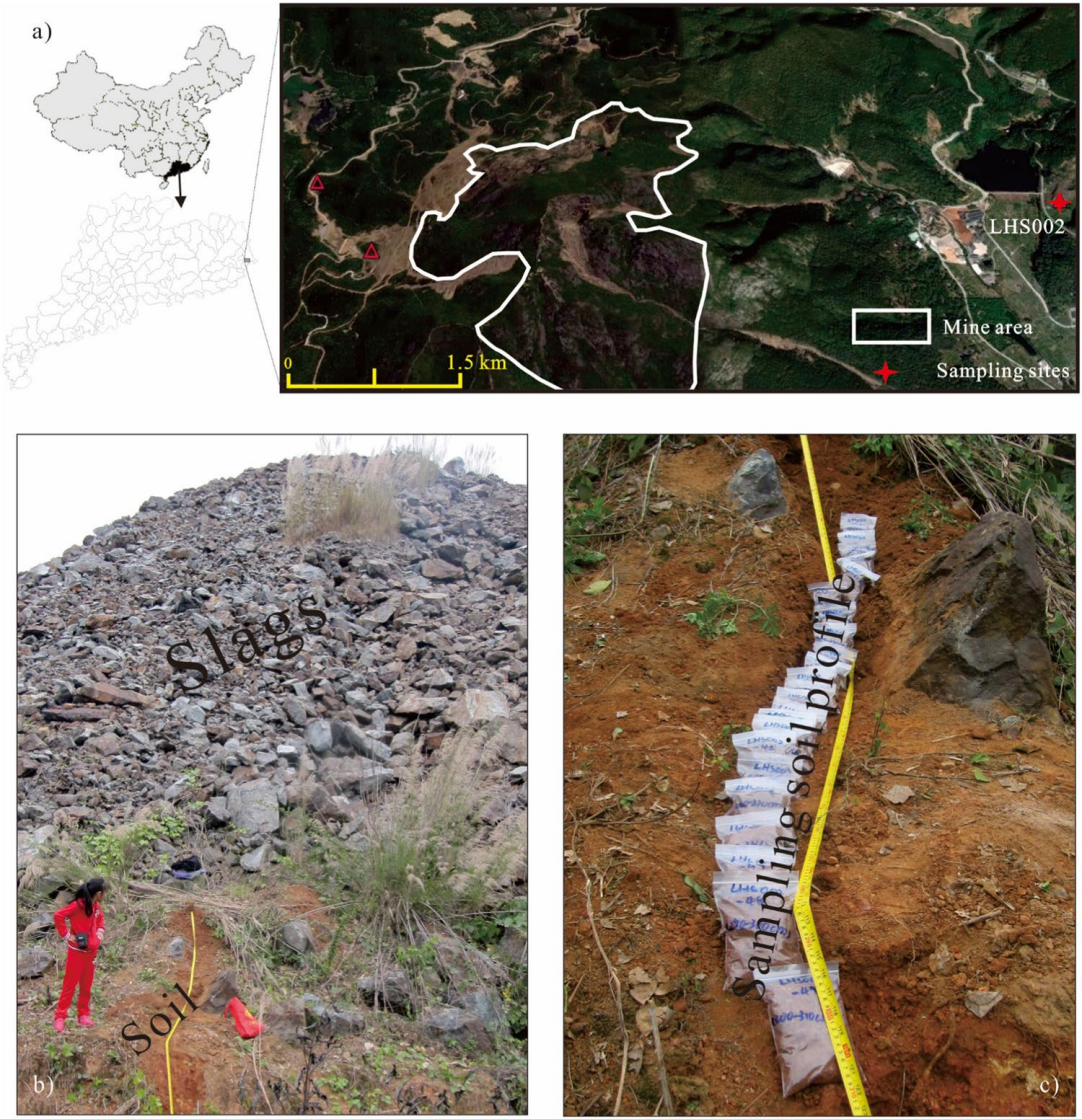
(**a**) Location of the study area and sampling site, (**b**) picture of the soil profile under slags, and (**c**) picture of collected soil samples. The maps in a) are generated based on the administrative maps of China and Guangdong Province using software ArcGIS (Version 10.2), which can be purchased from https://pan.baidu.com/s/17g_OCSbPKMaF7Vo4lvweYw.

A slag covered soil profile (about 310 cm sampling distance along a slope) located at about 2 km away from the mine area (Fig. 1a) was selected for magnetic investigation and arsenic content measurement. After digging out the profile, forty-nine soil samples were collected at 5 cm and 10 cm intervals from the upper 180 cm and below 180 cm, respectively. According to the terrain of the digged profile, the sections of upper 0–60 cm and lower 290–310 cm are vertical. Meanwhile, an about 30 degree of slope angle is measured for sampling distance 60–290 cm (Fig. 1b). Therefore, the actual sampling depth is 195 cm. From sampling depth 5 cm to 195 cm, forty-nine samples were collected from the profile (Fig. 1c). All samples were air-dried at room temperature and ground into a powder. The sample material was then packed into non-magnetic plastic cubes (8 cm^3^) for magnetic measurements. Subsamples were prepared for element content measurements by sieving through a 74 μm mesh.

### Magnetic and As content measurements

A suite of mineral magnetic analyses was performed for all samples at the Guangzhou Institute of Geochemistry, Chinese Academy of Sciences. Low (976 Hz) and high (15 616 Hz) frequency MS (mass-specific χ_lf_ and χ_hf_, respectively) were measured using a Kappabridge MFK1-FA (AGICO). Frequency-dependent MS was calculated from the expression χ_fd_ (%) = [(χ_lf_ − χ_hf_)/χ_lf_] × 100. Volume-specific MS as a function of temperature (κ vs T) was measured from room temperature to 700°C using a CS4 high-temperature unit attached to the MFK1-FA instrument. Anhysteretic remanent magnetization (ARM), expressed as the susceptibility of ARM (χ_ARM_), was imparted with a D-2000 (ASC Scientific Ltd.) alternating demagnetizer creating a 100 mT peak AF field and a DC biasing field of 0.05 mT. Isothermal remanent magnetization (IRM) acquisition curves with fifty-six steps up to 2500 mT were acquired using an IM-10–30 pulse magnetizer (ASC Scientific) and a JR6-A spinner magnetometer (AGICO) for intensity measurements (the JR6-A was also used for measuring ARM). Backfield IRM was imparted in a reverse 300 mT field. The IRM at an applied field of 2500 mT was regarded as saturation IRM (SIRM). An S-ratio S_−300_ was calculated using the equation [(-IRM_−300 mT_/SIRM) + 1]/2 according to Bloemendal *et al*.^31^. An estimate of the concentration of “hard” magnetic minerals (hematite and goethite) (HIRM) was calculated as HIRM = (SIRM + IRM_−300 mT_)/2^32^. The contribution of different magnetic components was quantified using the IRMUNMIX2_2 and IRM_CLG1 routines to analyze cumulative log-Gaussian (CLG) IRM acquisition curves^33,34^. Hysteresis parameters such as coercivity (Bc) and remanence coercivity (Bcr), which are helpful to identify the type and particle size of magnetic minerals^35,36^, were measured using a MicroMag 3900 VSM (Princeton Measurements), with a maximum applied field of 1.5T at Sun Yat-sen University.

The ground soil samples were dissolved in aqua regia according to the SUEPA standard method (3502)^37^ (USEPA, 1996). Then contents of arsenic (with detection limit of 0.2 mg/kg) as well as other trace elements were measured using an inductively coupled plasma mass spectrometry (ICP-MS, Agilent 7700×) at the ALS Minerals-ALS Chemex Laboratory, Guangzhou. The ALS is confident with the quality of the results since standard, duplicate, and blank samples were added during the measurement process followed by relevant specifications.

### Statistical analysis

Pearson bivariate correlation analysis was performed for various variables to reveal the relationship between magnetic properties and arsenic contents within the soil. In order to quantify the relationships of arsenic content and magnetic parameters, stepwise multiple regression analyses were performed using arsenic content and magnetic parameters as dependent and independent variables, respectively. Values of arsenic contents outliers outside of 2σ (σ: standard deviation) were eliminated from the regression analysis. All statistical analyses were carried out using SPSS 16.0 software.

## Results

### Magnetic mineralogy

**Figure 2:**
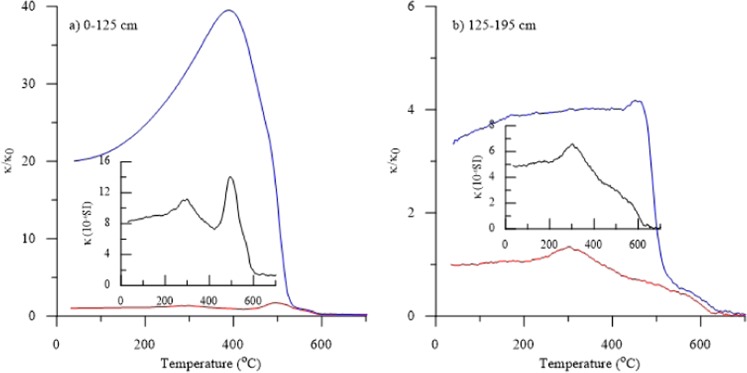
Thermal variation of magnetic susceptibility (κ-T curves) for representative samples from depths of (**a**) 0–125 cm and (**b**) 125–195 cm. Heating and cooling curves are shown in red and blue, respectively. The small inserts show enlargements of the heating curves.

Variation of MS with temperature (κ-T curves) is widely used to identify the type of magnetic minerals^10,11^. The dramatically increasing κ values (Fig. 2) after heating result from new formation of a magnetite-near phase, possibly due to the reaction of original hematite and clay minerals during high-temperature thermal treatment^38,39^ The neo-formation of magnetite is clearly stronger for samples from depths of 0–125 cm, and it already starts during heating at around 400 °C (Fig. 2a). For all samples along the entire profile, κ decreases between 270 °C and 400 °C (Fig. 2a,b), which can be interpreted as a signature of maghemite (γ-Fe_2_O_3_) inverting to weakly magnetic hematite (α-Fe_2_O_3_)^40,41^. The drop of κ in the heating curves around 580 °C (Fig. 2a,b) reveals the existence of magnetite in the initial samples, and the continuous decreasing κ above 600 °C for samples from depths of 125–195 cm (Fig. 2b) indicates a large contribution of hematite.

**Figure 3:**
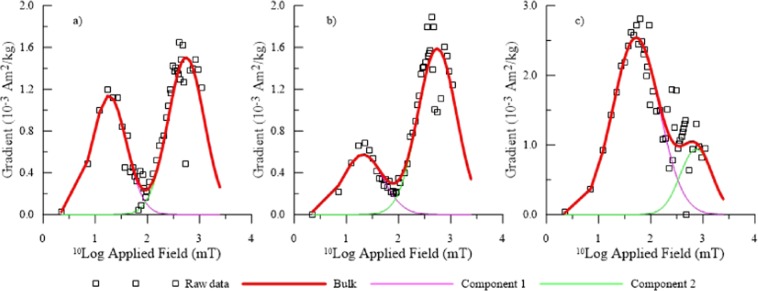
Gradient acquisition plots (GAP) of isothermal remanent magnetization (IRM) acquisition curves for representative soil samples from different depths, analyzed after Kruiver *et al*.^42^ using two components. (**a**) Approximately equal contributions of the two components to saturation IRM, (depth 0–30 cm); (**b**) Contribution of component 1 less than component 2 (depths 30–60 cm and 125–195 cm); (**c**) Contribution of component 1 larger than component 2 (depth 60–125 cm).

The CLG analysis results show a low coercivity and a high coercivity phase throughout the soil profile, but with different contributions (Fig. 3). The second component (C2) with B_1/2_ of several hundreds mT is likely related to hematite^42,43^. The low-coercivity component (C1) probably reflects the fraction of magnetite and maghemite as the existence of these phases is suggested by the shape of the κ-T curves.

### Vertical variation of magnetic properties and arsenic content

**Figure 4:**
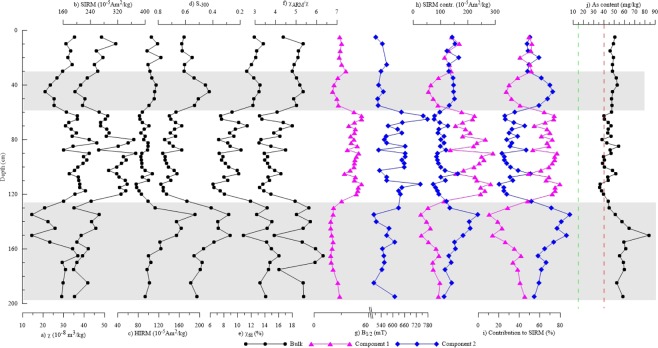
Vertical variation of magnetic properties and arsenic content. (**a**) Low filed magnetic susceptibility; (**b**) Saturation isothermal remanent magnetization; (**c**) Hard isothermal remanent magnetization; (**d**) S_−300_ ratio; (**e**) Frequency dependent magnetic susceptibility coefficient; (**f**) Ratio parameter susceptibility of anhysteretic remanent magnetization to low filed magnetic susceptibility; (**g**) B_1/2_ of different magnetic components; (**h**) SIRM contribution of different magnetic components; (**i**) Contribution of different magnetic components to SIRM; (**j**) Arsenic content. Green and red dashed lines represent the national first and secondary soil quality standards of the Chinese Environmental quality standard for soils (GB 15618-1995), respectively. Gray shades stand for sections that contribution of component 1 is less than component 2.

The statistical description and vertical variation of magnetic properties and the arsenic content are listed and illustrated at Table 1 and Fig. 4, respectively. According to the statistical arsenic contents listed in Table, the average valued of arsenic contents is 48.41 mg/kg which is higher than the value of the national secondary quality^44^, indicating a general soil arsenic pollution.

**Table 1:** Statistical summary of magnetic parameters and arsenic content.

Parameter	Range	Average ± SD
χ (10^−8^ m^3^/kg)	14.48–46.27	32.25 ± 6.91
SIRM (10^−5^ Am^2^/kg)	191.12–373.17	274.91 ± 52.23
HIRM (10^−5^ Am^2^/kg)	75.93–190.39	103.13 ± 22.68
Bc (mT)	7.96–61.78	15.96 ± 9.98
S_−300_	0.27–0.78	0.60 ± 0.14
χ_fd_ (%)	6.38–16.08	10.89 ± 2.72
χ_ARM_/χ	3.19–6.32	4.45 ± 0.86
IRM_C1 (10^−3^ Am^2^/kg)	0.28–2.92	1.55 ± 0.77
B_1/2__C1 (mT)	18.23–52.37	34.64 ± 11.34
Contribution to SIRM_C1 (%)	10.72–79.64	52.61 ± 18.95
IRM_C2 (10^−3^ Am^2^/kg)	0.72–2.37	1.24 ± 0.36
B_1/2__C2 (mT)	412.10–778.43	582.48 ± 74.36
Contribution to SIRM_C2 (%)	20.36–89.28	47.39 ± 18.95
As (mg/kg)	35.70–83.80	48.41 ± 8.64

The observed ranges of the parameters χ_lf_, SIRM, HIRM, IRM_C1, IRM_C2 indicate that total magnetic mineral concentration and the concentration of the two magnetic components largely change within the soil profile. The ranges of Bc, S_−300_, B_1/2__C1, and the SIRM contributions of C1 and C2 (Table 1) suggest a strong variation in the relative contents of the low and high coercivity magnetic components within the soil profile.

It can be seen from Fig. 4 that the magnetic mineral concentration-dependent parameters χ and SIRM show similar variations with higher and lower values in the upper (0–125 cm) and lower (125–195 cm) sections, respectively (Fig. 4a,b). The HIRM, which represents the concentration of high coercivity minerals, shows extreme high values at depths of 135–160 cm (Fig. 4c). The S_−300_, which reflects the relative concentration of low and high coercivity phases, is relatively high at depths of 0–30 cm and 60–120 cm, but very low at depths of 30–60 cm and 125–195 cm (Fig. 4d). The superparamagnetic (SP, ultrafine-grained status) particle sensitive parameter χ_fd_ and the ratio χ_ARM_/χ show similar variations with high-low-highest values at depths of 0–60 cm, 60–125 cm and 125–195 cm, respectively (Fig. 4e,f). The B_1/2_ of the low coercivity magnetic component C1 (magnetite and maghemite) ranges from 33–52 mT at depths of 60–120 cm, while it is clearly lower (<30 mT) in the upper and lower parts of the section (Fig. 4g). The contributions of the two magnetic components to the SIRM are relatively equal at depths of 0–30 cm. At depths of 30–60 cm and 125–195 cm, component C2 (hematite) dominates the SIRM, whereas at depths of 60–125 cm the contribution of C1 roughly doubles the one of C2 (Fig. 4h,i).

Arsenic contents show reverse changes (high and low values in the lower and upper parts of the section, respectively) with concentration-dependent magnetic parameters. Moreover, comparing to the “Chinese Environmental quality standard for soils (GB 15618–1995)^44^”, arsenic contents of only 7 samples among the collected 49 samples from the profile are below the national secondary quality, and arsenic contents of all samples exceed the standard of the first level (Fig. 4j).

### Relationship between arsenic content and magnetic properties

From the Pearson bivariate correlation coefficients listed in SI Table 1, significant negative correlation appears between the arsenic content and concentration-dependent parameters χ (mainly a measure of magnetite and maghemite contents) and SIRM (additionally includes the hematite content). A significant positive correlation with HIRM (a measure of hematite content), χ_ARM/_χ and Bc, while S_−300_ is negatively correlated (SI Table 1). This implies that the arsenic content is higher in samples with higher absolute hematite content and relatively more hematite contribution. A positive correlation with χ_fd_ indicates that the lower coercivity phase (magnetite and maghemite) has a finer grain size (reaching into the SP range) in samples with higher arsenic content.

## Discussion

### Mechanism of arsenic transportation and magnetic mineral transformation

**Figure 5:**
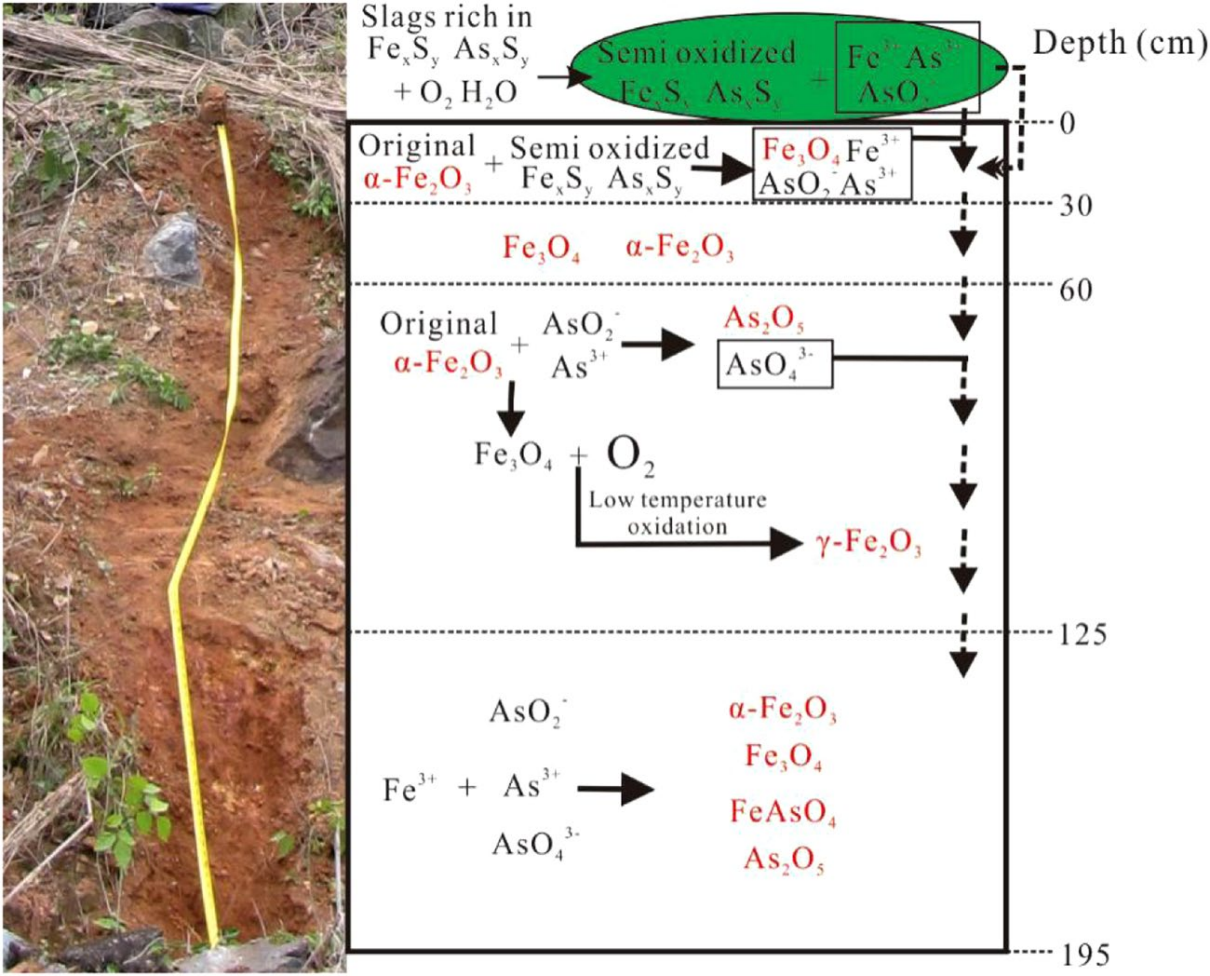
Sketch illustrating magnetic mineral transformation accompanied with arsenic transportation.

Compared to arsenic content of the national secondary quality^44^, the entire studied profile is moderately to seriously polluted by arsenic. Arsenic content increases from the upper to the lower part of the section (Fig. 4j). The high concentration of arsenic must derive from the overlying slags which are rich in arsenic sulfide, and the arsenic likely migrated into the soil due to rain washing. Naturally, magnetic mineral transformations in the form of maghemitization and hematization are generally occurred under tropical-subtropical hot and humid conditions during weathering processes^41^. Therefore, it can be speculated that maghemite and/or hematite dominated the sampled soil profile before slag coverage. After large amounts of slag rich in iron sulfides and arsenic sulfides were piled on the top, the magnetic mineral composition within the covered soils changed due to a large eluted of iron, arsenic, and sulfur from the slags^23,24^. The interpreted mechanism of magnetic mineral transformation and As transportation is illustrated at Fig. 5.

Under the hot and humid climate, the iron sulfides and arsenic sulfides were oxidized by Fe (III)^45^. During this oxidation process, iron and arsenic were transformed to movable iron and arsenic ion and were then transported from the upper to the lower part of the section^8^. With scouring of rain, the semi-oxidized iron sulfides and arsenic sulfides were drained into the uppermost part of the soil (depths of 0–30 cm) and then were further oxidized by Oxygen and the originally existing Fe_2_O_3_ (as oxidant) through the reaction As/Fe_x_S_y_ + O_2_/Fe_2_O_3_ = Fe_3_O_4_ + AsO_4_^3−^ + SO_4_^2−^. As a result, the contributions of magnetite and hematite to SIRM are approximately equal within this part of the soil profile (Fig. 4i). Part of the magnetite particles was translocated to a lower level (depths of 30–60 cm) within the section. The originally existing hematite within depths of 60–125 cm was reduced to magnetite and then partially transformed to maghematite through low temperature oxidation. Therefore, the contribution of magnetite and maghemite to SIRM is much higher than that of hematite at depths of 60–125 cm within the section. The oxidized arsenic migrated further down into a lower part (depths of 125–195 cm) in arsenic trioxide form and/or combined with Fe^3+^ to iron arsenate. These results indicate that the existing Fe_2_O_3_ in acid soils can enhance arsenic mobilization^21,46^ found that hematite has a stronger sorption capacity on arsenic than magnetite. Moreover, previous studies suggested that arsenic prefers to coexist with Fe_2_O_3_ in acid soils^27,47^. On the other hand, insoluble iron arsenate was generated and enriched in the profile together with higher hematite content. As a result, the highest arsenic content appears at depths of 135–155 cm in the sampled section with the highest content of hematite shown by largest HIRM values and the lowest values of the S_−300_ ratio (Fig. 4c,d).

### Quantification of soil arsenic content using magnetic parameters

As to the above discussion, transportation and the existing form of arsenic are closely related with the magnetic mineral parameters and the transformation of magnetic phases. According to the observed relationships, some easily available hematite-related parameters (e.g., HIRM, S_−300_) could be used to semi-quantify the arsenic content. This quantitative estimation may be also valid for other such areas.

The result of multiple regression analysis, based on 46 samples and omitting three samples with values of arsenic contents outside of 2σ, suggests that the soil arsenic contents can be best estimated from magnetic properties using the parameter S_−300_. As regression equation we obtain:

As (in mg/kg) = −50.22*S_−300_ + 78.754.

The normal P-P plot of the regression standardized residual (SI Fig. 1a), and values of the significance level (0.000) and adjusted R^2^ (0.618) indicate that this regression equation is effective for soil arsenic content quantification.

Using this regression equation and the measured S_−300_ values, predicted arsenic contents were calculated for the accepted 46 samples. A scatter plot of the predicted and measured arsenic contents is illustrated in SI Fig. 1b. All points distribute around the equilibrium line, further demonstrating the effectiveness of the regression equation for semi-quantification of soil arsenic contents.

## Conclusions

Magnetic mineral composition consists of magnetite/maghemite and hematite, with different contributions at depths of 125–195 cm, 60–125 cm, and 0–60 cm. The magnetic mineral composition results from pedogenesis and oxidation of iron and arsenic sulfides that were delivered from the slag cover.

The oxidation process changed part of the iron and arsenic sulfides to movable iron and arsenic ion, which were then transported from the upper to the lower part of the soil profile. As a result, arsenic pollution of the lower part of the soil profile is more serious than in the upper part.

The arsenic content shows a significant negative correlation with the concentration dependent magnetic parameters χ and SIRM, as well as with the hematite/magnetite(+maghemite) indicative ratio S_−300_. A significant positive correlation is found between the arsenic content and the absolute hematite concentration measured by the parameter HIRM. Soil arsenic may coexist with fine high coercivity hematite particles. The S_−300_ ratio is an effective choice for semi-quantification of soil arsenic content, which may be also used for soil arsenic monitoring in similar settings of arsenic polluted areas.

## Supplementary information

Supplementary information.
