# Supplementary material for: Magnetic response of Arsenic pollution in a slag covered soil profile close to an abandoned tungsten mine, southern China
Source: Sci Rep. 2020 Mar 9;10:4357. doi: 10.1038/s41598-020-61411-6 (PMC7062900; doi:10.1038/s41598-020-61411-6)
Supplement: Supplementary file 1 — Supplementary information. [file 41598_2020_61411_MOESM1_ESM.pdf]

Magnetic response of Arsenic pollution in a slag covered soil profile close to an abandoned tungsten mine, southern China

Tingping Ouyang[a, b*], Mingkun Li[a], Erwin Appel[c], Zhihua Tang[b, d], Shasha Peng[b], Sang Li[a], Zhaoyu Zhu[b]

[a] School of Geography, South China Normal University, Guangzhou 510631, China

[b] Key Laboratory of Ocean and Marginal Sea Geology, Guangzhou Institute of Geochemistry, Chinese Academy of Sciences, Guangzhou 510640, China

[c] Department of Geosciences, University of Tübingen, Hölderlinstrasse 12, 72074 Tübingen, Germany

[d] Guangzhou Institute of Energy Conversion, Chinese Academy of Sciences, Guangzhou 510640, China

[*]Corresponding author. Tel. +86 13632403778
Email address: oyangtp@m.scnu.edu.cn (Ouyang Tingping)

Supplementary Information

SI Figure 1 a) Normal P-P plot of regression standardized residual; b) Scatter plot between measured and predicted arsenic contents.

SI Table 1 Pearson correlation coefficients between arsenic content and magnetic parameters ($r_{0.001, 49} = 0.456$, $p<0.001$).

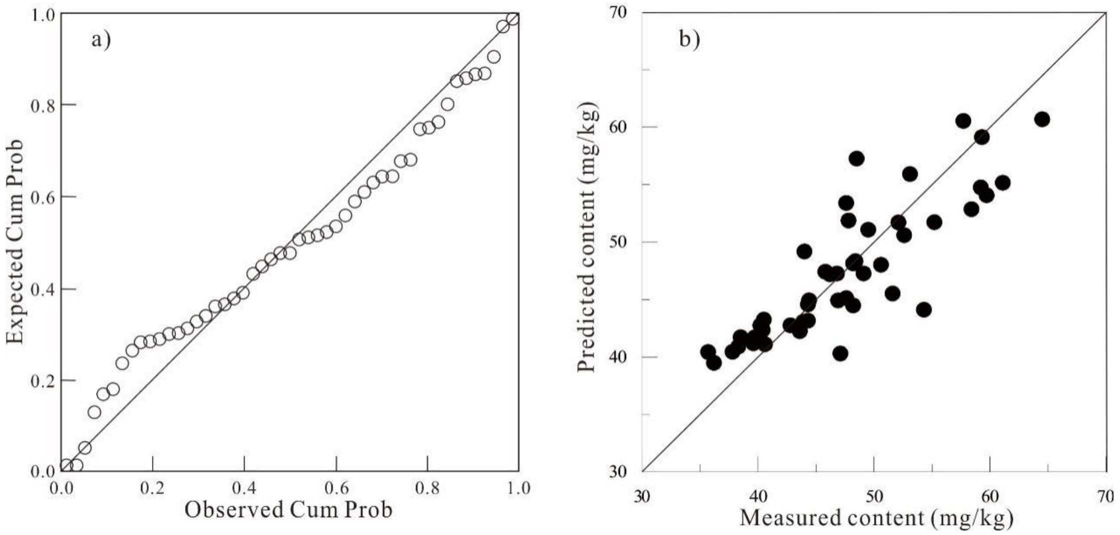

SI Figure 1 a) Normal P-P plot of regression standardized residual; b) Scatter plot between measured and predicted arsenic contents

SI Table 1 Pearson correlation coefficients between arsenic content and magnetic parameters ($r_{0.001, 49}$ = 0.456, p<0.001)

Parameters	χ ($10^{-8}m^3/kg$)	SIRM ($10^{-5}Am^2/kg$)	HIRM ($10^{-5}Am^2/kg$)	χ_{fd}%	χ_{ARM}/χ	S_{-300}	Bc (mT)
As (mg/kg)	-0.636	-0.636	0.677	0.666	0.544	-0.791	0.751
